# The association between EGF A61G polymorphism and risk of colorectal cancer in a Chinese population: a case-control study

**DOI:** 10.1042/BSR20190495

**Published:** 2019-05-15

**Authors:** Xiaoying Zhu, Yan Shen, Qigui Xie

**Affiliations:** 1 Department of Cancer daycare center, Sir Run Run Shaw Hospital, Zhejiang University School of Medicine, 3 Qingchun east Road, Hangzhou, Zhejiang, China; 2 Department of Gastroenterology, the Second Affiliated Hospital of Zhejiang Chinese Medical University, 318 Chaowang Road, Hangzhou, Zhejiang, China; 3 Department of Gastroenterology, Tongde Hospital of Zhejiang Province, 234 Gucui Road, Hangzhou, Zhejiang, China

**Keywords:** case-control study, colorectal cancer, EGF, Single nucleotide polymorphism

## Abstract

Epidermal Growth factor (EGF) could induce colorectal cancer (CRC) cell to develop epithelial mesenchymal-transition and enhance their ability to invade and migrate. Several studies have thrown light on the association between EGF gene polymorphism and risk of CRC, but with conflicting results. Therefore, we determined EGF A61G polymorphism by using PCR-restriction fragment length polymorphism method in 341 CRC cases and 472 controls in a Chinese population. Our results showed that EGF A61G polymorphism increased the risk of CRC in a Chinese population (GG vs AA: adjusted OR: 1.92; 95% CI: 1.27–2.91; *P*=0.002; GG+AG vs AA: adjusted OR: 1.43; 95% CI: 1.05–1.94; *P*=0.022; GG vs AG+AA: adjusted OR: 1.65; 95% CI: 1.15–2.39, *P*=0.007; G vs A: OR: 1.39; 95% CI: 1.14–1.69, *P*=0.001). Stratified analyses revealed that the significant association was more evident in the females, smokers, drinkers, and old subjects (age ≥60 years). Furthermore, the GG and/or AG genotype carriers were more likely to have larger tumor size and lymph node metastasis. In conclusion, EGF A61G polymorphism is a genetic contributor to CRC in a Chinese Han population.

## Introduction

Colorectal cancer (CRC) is the second leading cause of cancer deaths in Europe and the United States [1]. Previous data showed that 140,250 new cases of CRC were diagnosed in women and men, and 50,630 women and men died from this disorder during 2018 [2]. In China, CRC ranks the fifth and fourth most commonly diagnosed cancers amongst men and women respectively [3]. In addition, CRC is the fifth leading causes of cancer death amongst both men and women in China [3]. Up to date, the underlying mechanisms of CRC is still poorly understood. Studies have demonstrated that lifestyle, diet, and genetic factor might be associated with the susceptibility to CRC [4–6]. Genome-wide association studies (GWAS) have identified a host of novel gene loci for CRC patients.

Epidermal growth factor (EGF) is one of the most important cancer-related genes. It is located in chromosome 4q25. EGF via binding to its receptor (EGFR) is associated with survival, proliferation, and differentiation of epithelial cells [7]. Single nucleotide polymorphisms (SNPs) are the most common sources of human genetic variation, and they may be associated with cancer susceptibility [8]. EGF A61G polymorphism (a change of Guanine base [G] whit an Adenine base [A]) is one of the most important polymorphisms in EGF gene, located in the EGF 5′ UTR [9]. Studies have reported that EGF A61G polymorphism was associated with the EGF gene expression in normal colon in CRC patients [10]. Several studies [11–15] investigated the association between EGF A61G polymorphism and CRC risk; however, their findings were conflicting. In this case-control study, we aimed to evaluate the relationship between EGF A61G polymorphism and CRC susceptibility in a Chinese Han population.

## Patients and methods

### Subjects

Totally 341 patients diagnosed with CRC and 472 healthy controls were recruited from the Second Affiliated Hospital of Zhejiang Chinese Medical University (Hangzhou, China) and Tongde Hospital of Zhejiang Province (Hangzhou, China) from July 2013 to October 2018. The CRC patients were divided into two groups based on tumor location either colon (*n*=220) or rectum (*n*=121). Grading and staging of the tumor were classified. Tumor differentiation was grades as: well, moderately and poorly differentiated [16]. We excluded the subjects with family history of CRC and those who had received radiation therapy or chemotherapy. The cancer-free controls were selected from the individuals receiving health check-ups at these hospitals. Control subjects with malignant tumor or digestive system disease were excluded from the present study.

All participants signed a written consent and ethical committee of the above two hospitals approved all the ethical issues regarding the study. The demographic and risk factors were obtained through reviewing the medical sheet and questionnaires completed by all participants, including smoking status, drinking status, and family history of CRC.

### Blood sampling and genotyping

Genomic DNA was extracted from whole blood using the TIANamp Blood DNA kit (Tiangen Biotech, Beijing, China) according to manufacturer’s instructions. The quality and concentration of extracted DNA were evaluated using NanoDrop 2000 UV-VIS spectrophotometer (Thermo Scientific, U.S.A.).

EGF A61G polymorphism was analyzed using PCR–restriction fragment length polymorphism (PCR–RFLP). Based on the GenBank reference sequence, the PCR primers were as follows: forward 5’-GAG AAA CTG TTG GGA GAG GAA TC-3’ and reverse 5’-TCA CAG AGT TTA ACA GCC CTG C-3’. PCR for this SNP was carried out in a 25 μl reaction mixture containing 100 ng genomic DNA, 10 pmol of each primer, 10× PCR buffer, 50 mM MgCl_2_, 10 mM dNTP, and 0.5U Taq Polymerase. The PCR was performed under the following conditions: initial denaturation at 95°C for 4 min, followed by 30 cycles of 30 s at 94°C, annealing at 56°C for 30 s, polymerization at 72°C for 1 min, with a final polymerization step at 72°C for 10 min. The PCR product was digested with the endonuclease AluI (2 U at 37C for 3 h). Fragments were separated on a 3% agarose electrophoresis gel stained with ethidium bromide. The PCR product containing the 61*A/G allele produced 15, 34, 91, and 102 bp fragments or 15, 34, and 193 bp fragments respectively after digestion with AluI. To ensure the genotyping accuracy, 4% of selected samples were sent for direct sequencing.

### Statistical analysis

The demographic variables were expressed as means ± S.D. (continuous variables) and frequencies and percentages (categorical variables) respectively. The categorical data were compared using chi-square test and continuous data were compared using student’s *t* test and one-way ANOVA. Deviation between observed and expected frequencies amongst controls under the Hardy-Weinberg equilibrium (HWE) was analyzed using a goodness-of-fit chi-square test. The odds ratios (ORs) and corresponding 95% CIs for assessing the effect of the genotype distribution and allele frequencies of EGF A61G polymorphism on CRC were calculated by logistic regression analysis with adjustment for sex and age. The threshold for significance was *P*<0.05. All statistical analyses were conducted using SPSS 22.0 software (SPSS Inc., Chicago, U.S.A.).

## Results

### Characteristics of the study population

The demographic characteristics of participants are shown in Table 1. The mean age of CRC patients and healthy controls was 63.24 ± 7.53 and 62.24 ± 7.54 years, respectively. There is no significant difference amongst two groups with regard to sex, BMI, and smoking status. However, the distribution of drinkers amongst cases and controls differ significantly (*P*<0.001). Amongst the 341 cases, 208 (61.0%) were adenocarcinoma; 119 (34.9%) were squamous cell carcinoma; and 14 (4.1%) were other types of CRC (five were mucinous carcinoma; six were signet ring cell carcinoma, and three were undifferentiated carcinoma). The tumor stage for I, II, III, and IV were 70 (20.5%), 98 (28.8%), 101 (29.6%) and 72 (21.1%), respectively.

**Table 1:** Patient demographics and risk factors in CRC

Characteristics	Case (*n=*341)	Control (*n=*472)	*P*-value
Age	63.24 ± 7.53	62.24 ± 7.54	0.062
BMI	25.12 ± 1.46	25.17 ± 1.49	0.895
Sex			0.993
Male	68 (19.9%)	94 (19.92%)	
Female	273 (80.1%)	378 (80.08%)	
Smoking			0.177
Yes	189 (55.4%)	239 (50.64%)	
No	152 (44.6%)	233 (49.36%)	
Drinking			<0.001
Yes	252 (73.9%)	268 (56.8%)	
No	89 (26.1%)	204 (43.2%)	
Family history			
Yes	47 (13.8%)		
No	294 (86.2%)		
Histological grade			
Well differentiated	34 (10.0%)		
Moderately differentiated	265 (77.7%)		
Poorly differentiated	42 (12.3%)		
TNM stage			
I	70 (20.5%)		
II	98 (28.8%)		
III	101 (29.6%)		
IV	72 (21.1%)		
Tumor size			
>4 cm	197 (57.8%)		
≤4cm	144 (42.2%)		
Lymph node metastasis			
No	222 (65.1%)		
Yes	119 (34.9%)		
Location of CRC			
Colon cancer	220 (64.5%)		
Rectal cancer	121 (35.5%)		
Histology			
Adenocarcinoma	208 (61.0%)		
Squamous cell carcinoma	119 (34.9%)		
Others	14 (4.1%)		

### EGF gene A61G polymorphism and CRC susceptibility

The genotype and allele distributions for EGF gene A61G polymorphism amongst two groups are summarized in Table 2. The frequencies of AA, AG, and GG genotypes were 28.2, 49.7, and 22.1% respectively amongst the cases, and 36.6, 48.9, and 14.5% respectively amongst the controls. No significant deviation from HWE was found for A61G polymorphism in the control groups. The GG genotype had a 1.98-fold higher risk for CRC compared with AA genotype (GG vs AA: OR: 1.98; 95% CI: 1.31–2.99; *P*=0.001). Individuals with GG+GA genotypes were at higher risk than those carrying AA genotype (GG+AG vs AA: OR: 1.47; 95% CI: 1.09–1.99; *P*=0.012). EGF A61G polymorphisms was associated with the risk of CRC under the recessive and allelic models. Furthermore, the significant association also held true in additive, dominant and homozygous models after adjusting for sex, age, and alcohol consumption.

**Table 2:** Genotype frequencies of EGF gene rs4444903 polymorphisms in cases and controls

Models	Genotype	Case (n, %)	Control (n, %)	OR (95% CI)	*P*-value	*OR (95% CI)	*P-value
rs4444903							
Wild type	AA	96 (28.2%)	172 (36.6%)	1.00	-		
Heterozygote	AG	169 (49.7%)	230 (48.9%)	1.32 (0.96–1.82)	0.086	1.29 (0.93–1.79)	0.123
Homozygote	GG	75 (22.1%)	68 (14.5%)	**1.98 (1.31–2.99)**	**0.001**	** 1.79 (1.18–2.73)**	**0.006**
Co-dominant	AA vs AG vs GG				**0.007**		
Dominant	AA	96 (28.2%)	172 (36.6%)	1.00	-		
	GG+AG	244 (71.8%)	298 (63.4%)	**1.47 (1.09–1.99)**	**0.012**	**1.41 (1.04–1.92)**	**0.029**
Recessive	AG+AA	265 (77.9%)	402 (85.5%)	1.00	-		
	GG	75 (22.1%)	68 (14.5%)	**1.67 (1.16–2.40)**	**0.006**	**1.54 (1.06–2.23)**	**0.023**
Allele	A	361 (53.1%)	574 (61.1%)	1.00			
	G	319 (46.9%)	366 (38.9%)	**1.39 (1.14–1.69)**	**0.001**	-	-

The genotyping was successful in 340 cases and 470 controls for rs4444903.

* Adjust for age, sex, and alcohol consumption.

Furthermore, we conducted the stratified analyses to evaluate the effect of EGF A61G polymorphism on the risk of CRC according to sex, age, smoking, and alcohol (Table 3). Stratified analyses by sex indicated that EGF A61G correlated with the increased risk of CRC amongst female group (GG vs AA: OR: 1.87; 95% CI: 1.19–2.93; *P*=0.007), but not amongst male group (GG vs AA: OR: 2.76; 95% CI: 0.96–7.93; *P*=0.059). Similarly, a significantly increased CRC risk with the GG genotype was found in the smokers, drinkers, and old subjects (age ≥60 years).

**Table 3:** Stratified analyses between EGF rs4444903 polymorphisms and the risk of CRC

Variable	rs4444903 (case/control)			AG vs AA	GG vs AA	GG vs AA+AG	GG+AG vs AA
	AA	GA	GG				
Sex							
Male	19/35	37/51	12/8	1.34 (0.66–2.69); 0.417	2.76 (0.96–7.93); 0.059	2.30 (0.89–5.99); 0.087	1.53 (0.78–3.00); 0.217
Female	77/137	132/179	63/60	1.32 (0.92–1.89); 0.130	**1.87 (1.19–2.93); 0.007**	**1.58 (1.07–2.35); 0.023**	**1.46 (1.04–2.04); 0.029**
Smoking							
Yes	49/87	99/121	41/29	1.47 (0.94–2.27); 0.089	**2.51 (1.39–4.53); 0.002**	**1.98 (1.18–3.33); 0.010**	**1.67 (1.10–2.54); 0.017**
No	47/85	70/109	34/39	1.16 (0.73–1.85); 0.529	1.58 (0.88–2.82); 0.125	1.45 (0.87–2.42); 0.160	1.27 (0.82–1.97); 0.281
Drinking							
Yes	58/102	129/122	64/42	**1.88 (1.25–2.82); 0.003**	**2.68 (1.62–4.44); 0.001**	**1.82 (1.18–2.81); 0.007**	**2.08 (1.42–3.06); <0.001**
No	38/70	40/108	11/26	0.68 (0.40–1.17); 0.162	0.78 (0.35–1.75); 0.545	0.97 (0.46–2.05); 0.928	0.70 (0.42–1.17); 0.172
Age (years)							
<60	32/67	42/77	14/27	1.14 (0.65–2.01); 0.645	1.09 (0.50–2.35); 0.835	1.01 (0.50–2.04); 0.980	1.13 (0.66–1.92); 0.659
≥60	64/105	127/153	61/41	1.37 (0.93–2.03); 0.113	**2.44 (1.48–4.04); 0.001**	**2.00 (1.29–3.10); 0.002**	**1.60 (1.10–2.31); 0.013**

Bold values are statistically significant (*P*<0.05).

Subsequently, we analyze the role of A61G polymorphism in the clinicopathologic features of CRC patients (Table 4). GG genotype carriers were more likely to have larger tumor size (>4 cm/≤4 cm: GG vs AA: OR: 2.05; 95% CI: 1.07–3.94; *P*=0.039) and lymph node metastasis (yes/no: GG vs AA: OR: 1.69; 95% CI: 1.00–2.85; *P*=0.049) than AA genotype carriers.

**Table 4:** The associations between EGF rs4444903 polymorphism and clinical characteristics of CRC

Characteristics	Genotype distributions			
rs4444903	AA	AG	GG	GA+GG
Histological grade				
MD/WD	73/11	130/16	61/7	191/23
OR (95% CI); *P*-value	1.0 (reference)	1.22 (0.54–2.78); 0.628	1.31 (0.48–3.59); 0.595	1.25 (0.58–2.70); 0.566
Histological grade				
PD/WD	12/11	23/16	7/7	30/23
OR (95% CI); *P*-value	1.0 (reference)	1.32 (0.47–3.72); 0.602	0.92 (0.24–3.46); 0.898	1.20 (0.45–3.19); 0.721
TNM stage				
III+IV/I+II	51/45	86/83	36/39	122/122
OR (95% CI); *P*-value	1.0 (reference)	0.91 (0.55–1.51); 0.726	0.81 (0.45–1.49); 0.506	1.20 (0.45–3.19); 0.721
Tumor size				
>4 cm/≤4 cm	55/41	118/51	55/20	168/71
OR (95% CI); *P*-value	1.0 (reference)	**1.73 (1.02–2.90); 0.039**	**2.05 (1.07–3.94); 0.030**	**1.76 (1.08–2.88); 0.023**
Lymph node metastasis				
Yes/No	32/64	76/90	38/37	114/127
OR (95% CI); *P*-value	1.0 (reference)	**1.69 (1.00–2.85); 0.049**	**2.05 (1.10–3.82); 0.022**	**1.80 (1.10–2.94); 0.019**
Family history				
Yes/No	9/87	23/146	15/60	38/206
OR (95% CI); *P*-value	1.0 (reference)	1.52 (0.67–3.44); 0.309	2.42 (0.99–5.88); 0.074	1.78 (0.83–3.85); 0.136
Histology				
Adenocarcinoma/Not	60/36	98/71	49/26	147/97
OR (95% CI); *P*-value	1.0 (reference)	0.83 (0.50–1.38); 0.472	1.13 (0.60–2.12); 0.702	0.91 (0.56–1.48); 0.701
Location of CRC				
Colon cancer/Rectal cancer	59/37	109/60	51/24	160/84
OR (95% CI); *P*-value	1.0 (reference)	1.14 (0.68–1.91); 0.622	1.33 (0.71–2.52); 0.376	1.20 (0.73–1.95); 0.476

Bold values are statistically significant (*P*<0.05). MD, moderately differentiation; PD; poorly differentiation; WD; well differentiation.

## Discussion

In the present study, we found that EGF A61G polymorphism was associated with the increased risk for CRC, especially amongst female, old subjects (age ≥60 years), smokers, and drinkers. Furthermore, A61G polymorphism showed significant correlation with tumor size and lymph node metastasis in CRC patients.

Wu et al. conducted a case-control study to explore the relationship between EGF A61G polymorphism and CRC risk in a Germany population [11]. They found that EGF A61G polymorphism was associated with increased risk for CRC [11]. In addition, they suggested that EGF 61 G/G genotype and the G allele were related to CRC susceptibility [11]. However, they [11] obtained no association between this SNP and the tumor stages or the tumor grading of CRC. In a subsequent study, Lin et al. replicated positive findings in a Chinese Han population [13]. They showed that EGF 61 G/G genotype was associated with a higher risk of colon cancer, but not rectal cancer [13]. However, the present study showed that EGF A61G polymorphism was a risk factor for CRC, but not only for colon cancer. In the subgroup analyses of tumor size, tumor location, differentiation, growth pattern, and TNM stage of colon cancer, Lin et al. obtained no significant results [13]. In the present study, we observed EGF A61G polymorphism was related to the tumor size and lymph node metastasis in CRC patients, indicating that this SNP may be a diagnostic marker for CRC patients with bigger tumor size and lymph node metastasis. It is obvious that the findings of the present study were not consistent with those of the study by Lin et al. [13]. We assumed several factors may contribute to these disaccords. First, the present study indicated that there were some interactions between EGF A61G polymorphism and some environment factors including smoking and drinking; while Lin et al. [13] did not find this. Second, Lin et al. [13] obtained significant findings in colon cancer, while the present study yielded positive association in CRC. Third, the present study enrolled the population from Eastern China while Lin et al. [13] recruited study population from Northern China. Obviously, the living environments and eating habits may potential reasons for these inconsistences. In addition, several studies from other populations from Iran [12,14] and Malaysia [15] did not observe significant association between EGF A61G polymorphism and CRC risk. Ethnic heterogeneity, clinical heterogeneity, diverse genotype distributions, different sample sizes, eating habits, and different exposure factors may contribute to the discrepancies of these abovementioned studies. In the present study, we found that EGF A61G polymorphism was related to increased risk for CRC in this Chinese Han population. Several meta-analyses [17–19] validated our findings and showed this SNP was associated with increased risk for CRC. In addition, stratified analyses revealed that EGF A61G polymorphism was associated with the increased risk of CRC amongst the females, old subjects (age ≥60 years), smokers, and drinkers, indicating that the interactions between those factors and EGF A61G polymorphism contributed to increased risk for CRC patients.

Previous studies showed that EGF 61 gene polymorphism has a functional influence on EGF gene expression in normal colon in CRC patients [10]. Furthermore, GG genotype was reported to be associated with more EGF gene expression [9,10]. In the present study, we found GG genotype carriers were more prone to the occurrence of CRC. Thus, we assumed that GG genotype of A61G polymorphism may increase the EGF production, thereby contributing to increased risk for CRC.

For the present study, several limitations need to be addressed. First, the sample size of the present study was not large. Second, the interaction between environmental factors and genetic factors should be explored. Third, selection bias was inevitable because this is a retrospective study. Fourth, only one SNP was investigated in the present study. At last, because of the restriction to Chinese descent, these findings should be verified in other ethnicities.

To sum up, the present study suggested that EGF A61G polymorphism is associated with increased risk for CRC in a Chinese population. Further studies in other studies with larger sample sizes amongst Chinese Han population should be performed in future.

## Abbreviations

CRC: colorectal cancer
EGF: epidermal growth factor
GWAS: genome-wide association studies
HWE: Hardy-Weinberg equilibrium
OR: odds ratio
SSNP: single nucleotide polymorphism
